# Results of Cervical Recapping Laminoplasty: Gross Anatomical Changes, Biomechanical Evaluation at Different Time Points and Degrees of Level Involvement

**DOI:** 10.1371/journal.pone.0100689

**Published:** 2014-06-20

**Authors:** Yu Si, Zhenyu Wang, Tao Yu, Guo zhong Lin, Jia Zhang, Kuo Zhang, Hua Zhang, Yuan chao Li

**Affiliations:** 1 Department of Neurosurgery, Peking University Third Hospital, Beijing, People’s Republic of China; 2 Department of Laboratory Animal Science, Beijing, People’s Republic of China; 3 Clinical Epidemiology Research Center, Peking University Third Hospital, Beijing, People’s Republic of China; 4 School of Mechanical Engineering,Shanghai JiaoTong University, Shanghai, People’s Republic of China; University of Toronto, Canada

## Abstract

**Background:**

Recapping laminoplasty has become the frequently-used approach to the spinal canal when bone decompression of the vertebral canal is not the goal. However, what changes will occur after surgery, and whether recapping laminoplasty can actually reduce the risk of delayed deformities remains unknown.

**Methodology:**

We designed an animal experiment using a caprine model, and partitioned the animals into in vitro and in vivo surgical groups. We performed recapping laminoplasty on one group and laminectomy on another group. These animals were sacrificed six months after operating, cervical spines removed, biomechanically tested, and these data were compared to determine whether the recapping laminoplasty technique leads to subsequent differences in range of motion. Image data were also obtained before the surgery and when the animals were killed. Besides, we investigated the initial differences in kinetics between recapping laminoplasty and laminectomy. We did this by comparing data obtained from biomechanical testing of in vitro-performed recapping laminoplasty and laminectomy. Finally, we investigated the effect that longitudinal distance has on cervical mechanics. This was determined by performing a two-level recapping laminoplasty, and then extending the laminoplasty to the next level and repeating the mechanical testing at each step.

**Principal Findings:**

There were three mainly morphological changes at the six months after laminoplasty: volume reduction and bone nonunion of the recapping laminae, irregular fibrosis formation around the facet joints and re-implanted lamina-ligamentous complex. In the biomechanical test, comparing with laminectomy, recapping laminoplasty didn’t show significant differences in the immediate postoperative comparison, while recapping laminoplasty demonstrated significantly decreased motion in flexion/extension six months later. Inclusion of additional levels in the laminotomy procedure didn’t lead to changes in immediate biomechanics.

**Conclusions:**

Recapping laminoplasty can’t fully restore the posterior structure, but still reduced the risk of delayed cervical instability in a caprine model.

## Introduction

When treating spinal cord tumors, excision via laminectomy is an classic surgical intervention in practice, but many clinicians have reported that its longitudinal results can be less than ideal [1], [2], [3], [4], [5]. In order to avoid these drawbacks, a variety of laminoplasty methods have been described since the late 1970s. The most two popular and adequately-studied laminoplasty techniques are “open door laminoplasty” and “french door laminoplasty”. Both these two procedures involve a “door” technique, incising one side of the lamina while hinging to the contralateral side. So, these techniques inevitably obscure dural exposure. When treating spinal cord tumors and vascular malformations, these limitations are more obvious [6], [7]. So, they are all unsatisfactory choices when operators deal with spinal cord lesions.

In this case, recapping laminoplasty is a potentially valuable option [7], [8]. The purported advantage of this new technique is structure recovery of the rear bone tissue, which, theoretically, produces a return to more normal biomechanics of the spine. Meanwhile, recapping laminoplasty allows more wide intradural access. So, recapping laminoplasty is applicable to be the approach to the spinal canal when bone decompression of the vertebral canal is not the goal of the surgical procedure. However, which changes will occur after surgery, and whether recapping laminoplasty can actually reduce the risk of delayed deformities remains unknown to us. And, we are not aware of any anatomical or biomechanical study that has attempted to test this hypothesis.

Most of the available biomechanical literatures involving various surgical techniques have compared immediate resultant spinal motion and alignment patterns in human cadavers [9], [10]. However, this method has limitations: it can’t simulate the postoperative changes in the body, especially for recapping laminoplasty. Because there are two distinct states whether the fusion of the laminae are achieved or not, and this difference may significantly affect the stability after surgery. So, we designed an animal experiment using a caprine model, and compared with laminectomy in the early and late stage respectively.

In addition, the number of intervertebral levels involved in a given recapping laminoplasty procedure varies depending on the degree of lesion involvement. For example, Andrea Ruggeri retrospectively reviewed 40 patients and reported that they operated on an average of 2 levels (range: one to six levels) [6]. Whether extending the recapping laminoplasty will deliver undue instability to the spine is also a question clinicians concerned.

In order to answer these questions as well as provide clinically useful informations, we chosen cervical vertebra as the research object, and identified three specific aims: (1) to observe the morphological changes after cervical recapping laminoplasty by using imaging techniques; (2) to measure the initial and six-months differences in kinetics between the cervical recapping laminoplasty and laminectomy techniques; and (3) to determine whether inclusion of additional levels in the recapping laminoplasty procedure results in a change in immediate, postoperative cervical biomechanics.

## Materials and Methods

The goat model was selected because such models have been extensively accepted for evaluation of the human cervical biomechanics and the associated longitudinal changes by previous investigators [11], [12], [13], [14]. Similarities were seen between goats and humans with respect to the size, morphologic characteristics, alignment of the facet joints, upright head–neck positions and the cervical curvature (lordosis).

In order to achieve the intended goal of the experiment, we partitioned the animals into in vitro and in vivo surgical groups. We performed recapping laminoplasty on one group and laminectomy on another group of animals. These animals were sacrificed 6 months after operating, cervical spines removed, biomechanically tested, and these data were compared to determine whether the recapping laminoplasty technique leads to subsequent differences in range of motion of the cervical spines. Image data were also obtained before surgery and when the animals were killed. In addition, we investigated the initial differences in kinetics between the cervical recapping laminoplasty and laminectomy. We did this by comparing data obtained from biomechanical testing of in vitro-performed recapping laminoplasty and laminectomy. Finally, we investigated the effect that longitudinal distance has on cervical mechanics. This was determined by performing a two-level recapping laminoplasty on harvested spines, mechanically evaluating the spines, and then extending the recapping laminoplasty to the next level and repeating the mechanical testing. The following describes these procedures in greater detail. The mechanical testing protocol was the same for all three studies and is also described below.

### Ethics Statement

This investigation followed international guidelines (Guide for the Care and Use of Laboratory Animals) for protection of animals and was approved by the ethics committee of the Peking University. Sample preparation.

The titanium screws and plates are Johnson & Johnson’s products, and have been widely used in clinical practice.

### Surgical Techniques

#### Recapping laminoplasty versus laminectomy (six months postoperatively)

The recapping laminoplasty versus laminectomy investigation included three different animal groups (n = 7 for each group): intact (INT), recapping laminoplasty, and laminectomy. The surgical segments of recapping laminoplasty and laminectomy are both from C3 to C6. We did not observe neurologic changes after any of the surgeries.

#### Intact group

We tested seven normal caprine spines in order to determine the normal load-displacement characteristics of the caprine cervical spine. Animals were sacrificed and their cervical spines immediately harvested, wrapped in saline-soaked gauze, placed in sealed double plastic bags, and frozen at −80°C until ready for mechanical testing.

#### Recapping laminoplasty

General anesthesia was administered with the goats in a prone position. An incision was made from C2 to T1. The nuchal ligament was midline incised, and the perispinous muscles were subperiosteally dissected from the spinous processes to the medial margins of the facets. Care was taken not to detach the paraspinal muscle from C2 in order to maintain an effective extensor force and preserve the overall neck alignment. A high-speed drill was used to cut the laminae caudo-cranially following a line which passes halfway between each spinous process and zygapophysial joint, obviously preserving its articular capsule, from C3 to C6. The interspinous ligaments at rostral-most and caudal-most levels of the recapping laminoplasty segments were cut and the flap was elevated. The osteo-ligamentous complex was therefore formed by the laminae, the supraspinous, the yellow and the interspinous ligaments. It was stored in 250 cc of saline solution plus Gentamicin 80 mg/2 ml. Reconstruction was done using titanium plates and screws. Attention must be paid to correctly shape the plates and to fix them in order not to violate the zygapophysial joints. Absorbable vicryl sutures reconnected the supraspinous and interspinous ligament. Other more superficial tissues were sutured as usual. All animals were sacrificed 6 months postoperatively via injection of sodium pentobarbital. The spines were immediately harvested, wrapped in saline-soaked gauze, sealed in double plastic bags and stored at −20°C until ready for testing.

#### Laminectomy

Precisely, it should be “facet-sparing laminectomy”. For this group, the incision of the posterior longitudinal skin and nuchal ligament, and muscle dissection were done according to the same technique described above. Next, a four-level laminectomy was performed from C3 to C6 levels using a high-speed pneumatic drill and assorted Kerrison rongeurs. The spinous processes and laminae were removed following a line which passes halfway between each spinous process and zygapophysial joints, leaving a narrow channel exposing the spinal canal. The lamina and ligamentum flavum were resected while the integrity of the facet joint was maintained. All animals were sacrificed 6 months postoperatively via injection of sodium pentobarbital. The spines were immediately harvested, wrapped in saline-soaked gauze, sealed in double plastic bags and stored at −80°C until ready for testing.

#### Animal care

After the operation, the animals were fed a regular diet and allowed to bear their full weight and received antibiotics (penicillin, 80,000 U/d) for 3 days.

### Longitudinal Effects of Recapping Laminoplasty

**Figure 1:**
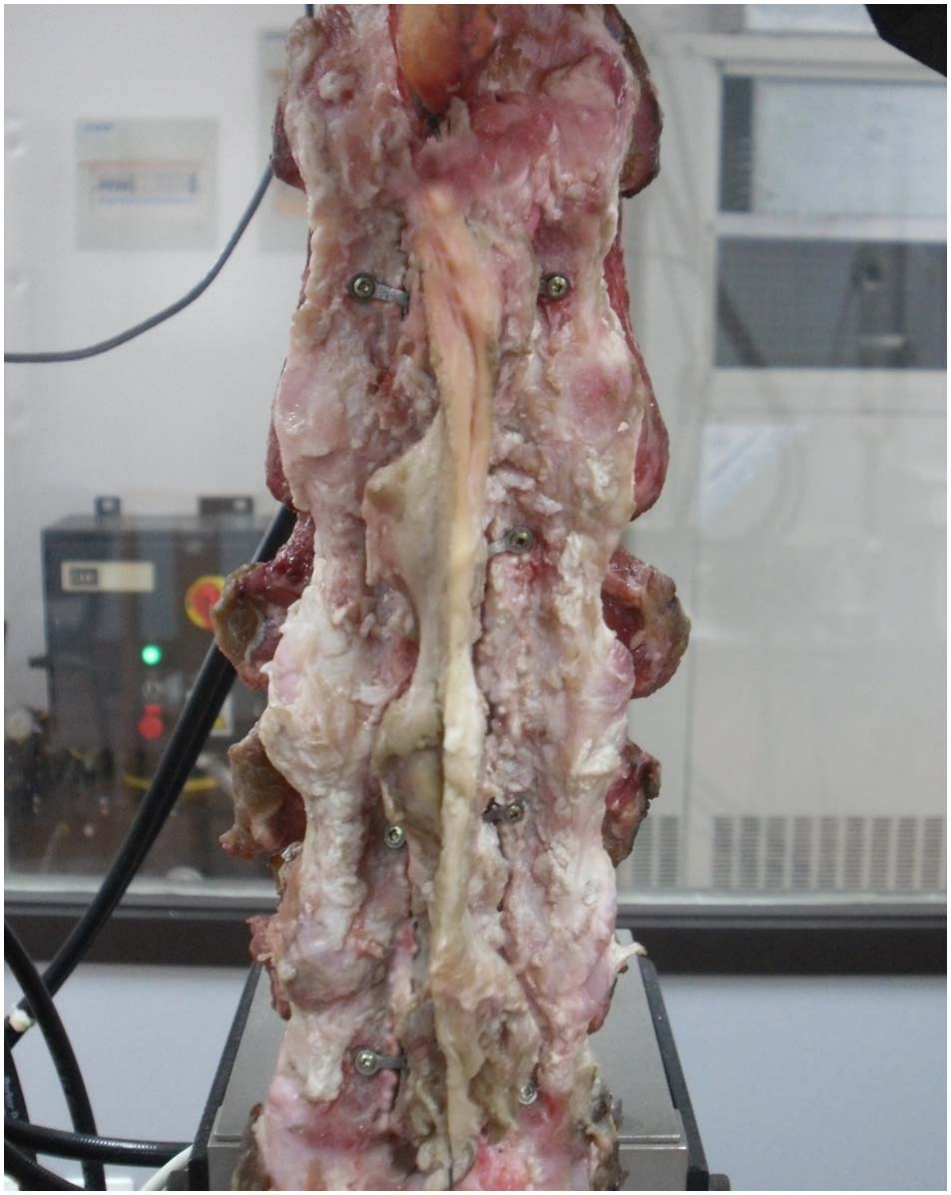
The four-level caprine recapping laminoplasty model. The osteo-ligamentous complex was reapproximated and attached with titanium microplates.

We tested seven normal caprine spines in order to determine the normal load-displacement characteristics of the goat cervical spine (INT group). The recapping laminoplasty technique (as described above) was performed in vitro for all of these procedures (Fig. 1). Each specimen was tested in the following order:

- INT: intact without recapping laminoplasty.
- 2L: two-level recapping laminoplasty (C3–C4).
- 3L: three-level recapping laminoplasty (C3–C5).
- 4L: four-level recapping laminoplasty (C3–C6).

### Initial Differences between Recapping Laminoplasty and Laminectomy

Between 3L and 4L, we tested the biomechanical characteristics of four-level laminectomy (C3–C6). Specifically, we compared the 4L data from the longitudinal effects of recapping laminoplasty with the data of four-level laminectomy.

### Biomechanical Testing Protocol

**Figure 2:**
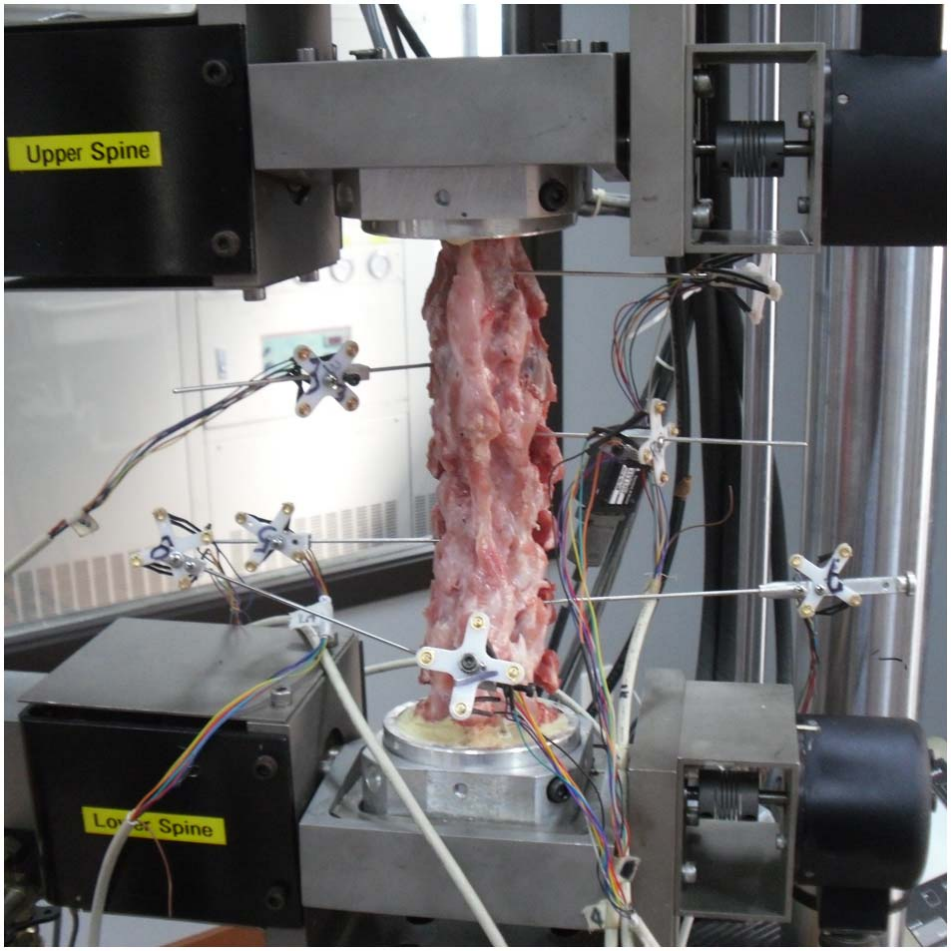
Biomechanical test. Photograph depicting the experimental apparatus. both ends of the cervical spine were embedded, and mounted to a MTS multifunctional mechanical testing machine. The motion of the sensors was tracked with an optical motion capture system.

On the day of testing, the specimens were thawed to room temperature. The skin and superficial muscular tissues were dissected, leaving sufficient muscles to keep the ligamentous tissues intact and moist during the specimen preparation and testing. The superior half of the C2 vertebra and inferior half of the T1 vertebra were embedded with polymethyl-methacrylate (PMMA), and mounted to a servo hydraulic materials testing machine (858Bionix II; MTS Corporation, Eden Prairie, MN) retrofitted with two initiative-driven fixtures. The fixture was two axis controlled spine kinematics sub-system (858Bionix II; MTS Corporation, Eden Prairie, MN). Its two axes of rotation were driven respectively by two servo-hydraulic cylinders and finally achieve the measurement and control of torque and angle. The fixture shared the same control system with Servo hydraulic materials testing machine. Custom-made rigid body sensors consisting of 4 IREDs were rigidly attached to the anterior of each vertebra and the upper and lower gimbals. The motion of the sensors was tracked with an optical motion capture system (Optotrak 3020; Northern Digital Inc., Waterloo, Ontario, Canada) (Fig. 2).

The machine applied a constant load (30 N) in the axial direction before testing. Each test included pure moment loading (±2 N·m) in flexion/extension, right/left lateral bending, and right/left axial rotation at a loading rate of 2 N·m/min. To precondition the specimen and to minimize the viscoelastic effects, each loading cycle was repeated 3 times, with the data from the third cycle used for analysis. The specimens were copiously moistened using 0.9% sodium chloride irrigation solution throughout the study.

### Imaging Examination

Computed tomography inspections of the cervical spines were performed before the surgery and sacrifice of each animal. After biomechanical tests, each single targeted vertebra of recapping laminoplasty group was harvested, and then examined by micro-CT scanner (Explore Locus SP, GE Healthcare) using a voxel size of 14-mm in all three axes. The titanium screws and plates were removed now in order to avoid the formation of artifacts. The images of the bone were then obtained and reconstructed.

### Statistics

The data were presented as the mean±SD. For the longitudinal and initial effects study, data were analyzed using a repeated-measures, one-way analysis of variance (p<0.05 significance). For the six-months effect study, data were analyzed using a one-way analysis of variance (p<0.05 significance). Individual differences between each group was delineated with Tukey’s post-hoc test (p<0.05 significance level). Statistical analyses were performed using SPSS 15.0 statistical software (SPSS Inc., Chicago, IL, USA).

## Results

### Anatomical Changes

**Figure 3:**
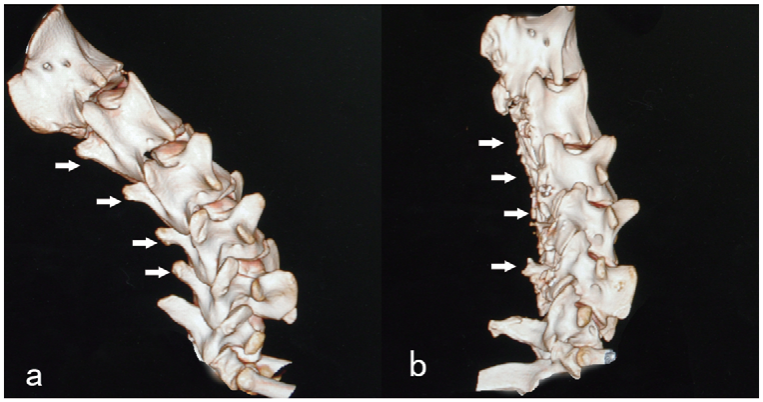
CT reconstruction of the cervical spine. a show the preoperative picture of the spinous process (C3–C6, arrows); b show the picture of the spinous process six months after surgery, the spinous process of the surgical vertebra is almost gone (C3–C6, arrows).

**Figure 4:**
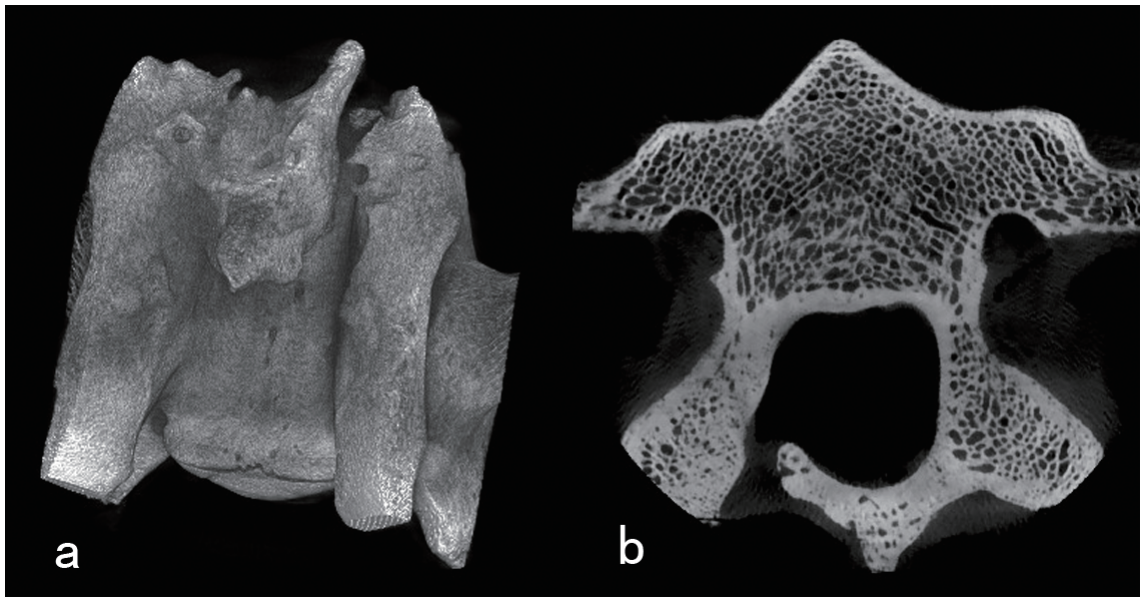
Micro-CT examination of the postoperative vertebra six months after surgery. a demonstrate the unilateral fusion of the lamina, and the length of the lamina is also minished. b demonstrate the bone density of the residual lamina was visually almost the same as the vertebral arch with no intervention.

**Figure 5:**
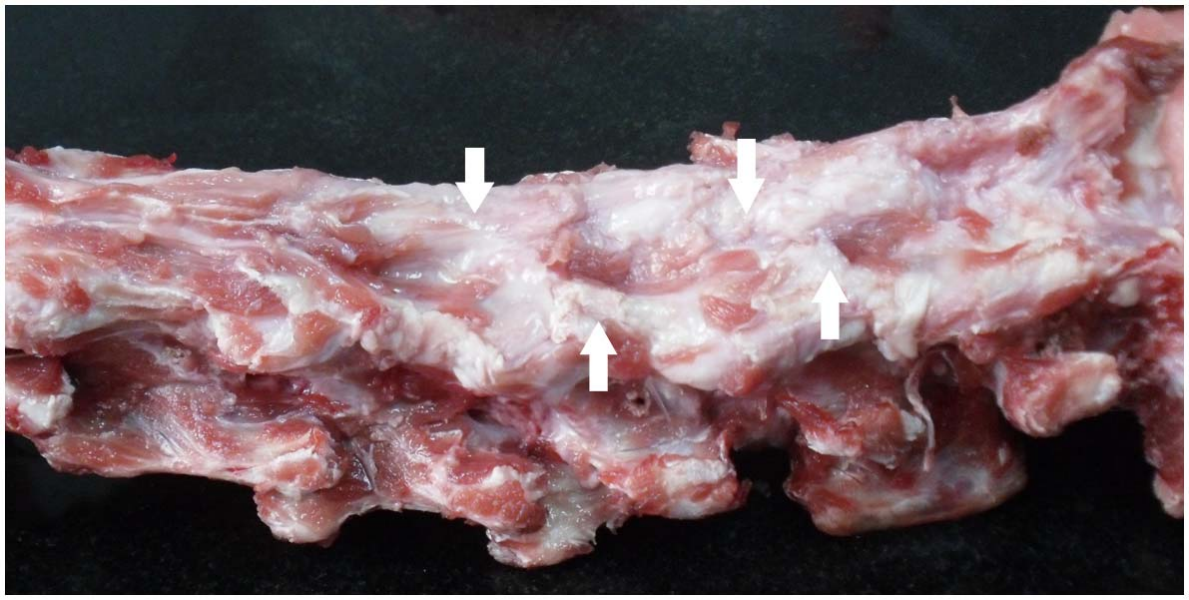
Gross anatomy performance of the cervical spine six months after surgery. Photograph depict appreciable abundant fibrous tissue formation round the facet joints and re-implanted lamina-ligamentous complex (arrows).

1, the three-dimensional spiral CT reconstruction depicted the significantly volume reduction of the recapping lamina: the spinous processes were shortened, and the length of the lamina was also reduced (Fig. 3). 2, in summary, twenty-eight laminae were re-implanted in 7 goats. Bilateral healing of the incision lines was observed in eighteen cases, unilateral healing in seven cases, non-healing in three cases. No evidence of “cross-level” fusion occurs in either group in our experiment. The Micro-CT examination showed the unilateral fusion of the lamina. The length of the lamina has been minished. However, the bone density of the residual lamina was visually almost the same as the vertebral arch with no intervention (Fig. 4). 3, the gross specimens demonstrated abundantly irregular fibrosis formation around the facet joints and re-implanted lamina-ligamentous complex (Fig. 5).

### Biomechanical Evaluation

#### Recapping laminoplasty versus laminectomy (six months postoperatively)

**Figure 6:**
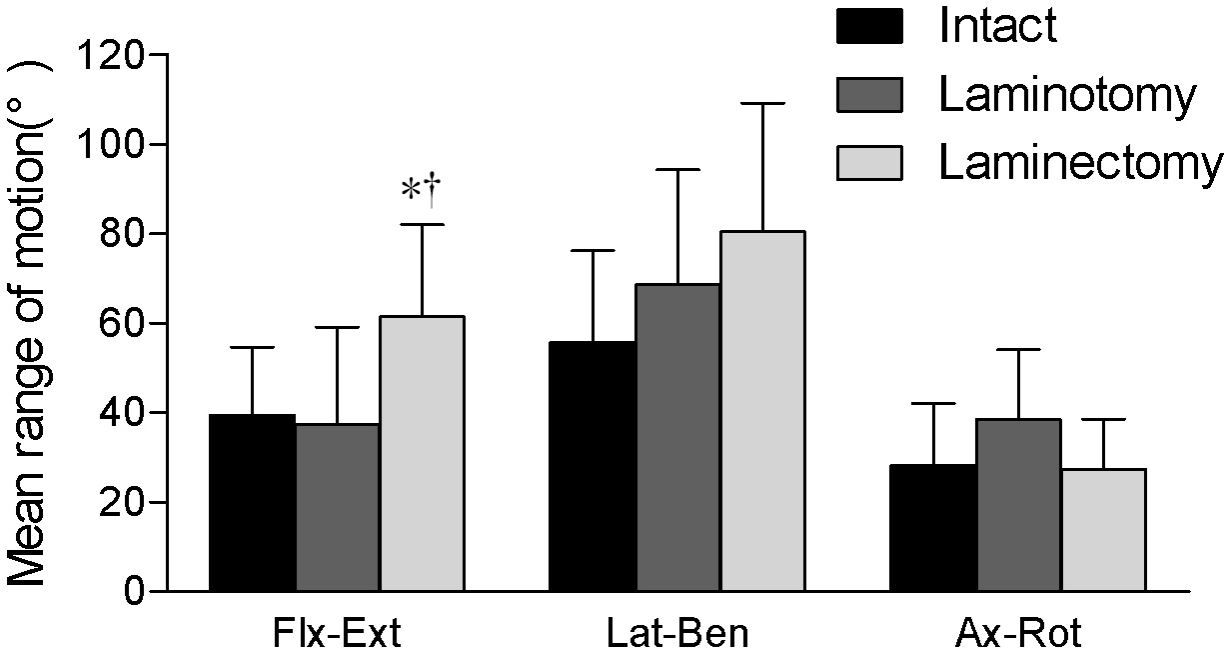
Recapping laminoplasty versus laminectomy (six months postoperatively). The range of motion (with one standard deviation error bar) for the INT, recapping laminoplasty (abbreviated as laminoplasty) and laminectomy conditions for the three rotation planes. *Significant difference between intact and laminectomy. †Significant difference between Recapping laminoplasty and laminectomy.

Range of motion data are shown in Fig. 6. The flexion/extension data indicated that laminectomy (61.5°±20.5°) significantly increased sagittal-plane motion compared with the INT condition (39.5°±15.1°) and the recapping laminoplasty technique (37.4°±21.7°). The difference between other surgical groups was not statistically significant.

#### Longitudinal effects of recapping laminoplasty

**Figure 7:**
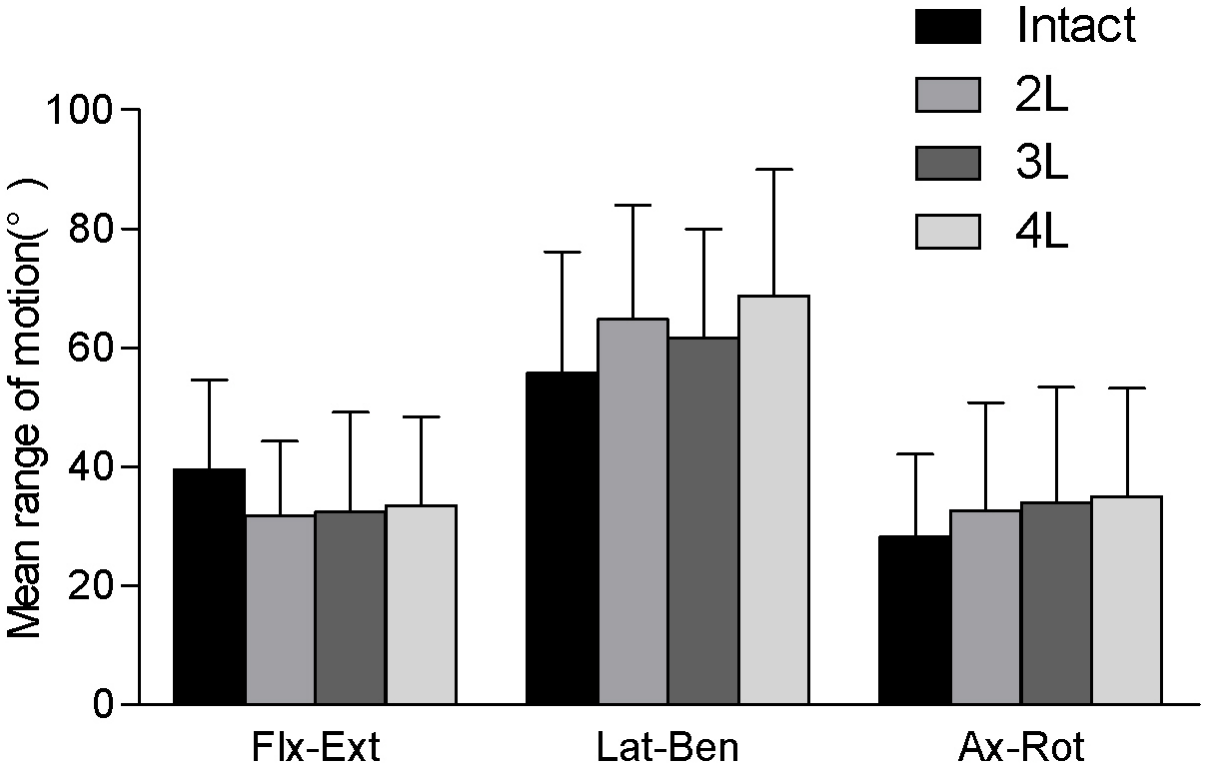
Longitudinal effects of recapping laminoplasty. The range of motion (with one standard deviation error bar) for all four model conditions. There was no statistical difference (p>0.05) in motion for any condition.

Overall, our data indicated equivalence between the intact spine and the three different recapping laminoplasty conditions (Fig. 7). In flexion–extension, the INT condition demonstrated the greatest C2–T1 motion (39.5±15.1°) and the 2L condition allowed the least motion (31.7±12.6°). There were no statistically significant differences between any of the groups in flexion–extension. Axial rotation C2–T1 ROM ranged from 28.2° (INT condition) to 35.0° (4L condition), with each group being statistically equivalent. Finally, lateral bending demonstrated the greatest amount of motion for all groups. The INT condition showed the least amount of C2–T1 lateral bending ROM (55.7±20.5°), and the 4L condition showed the highest degree of lateral bending ROM (68.8±21.1°). Once again, repeated-measures ANOVA indicated that there were no statistically signify cant differences between any of these groups in lateral bending.

#### Initial differences between recapping laminoplasty and laminectomy

**Figure 8:**
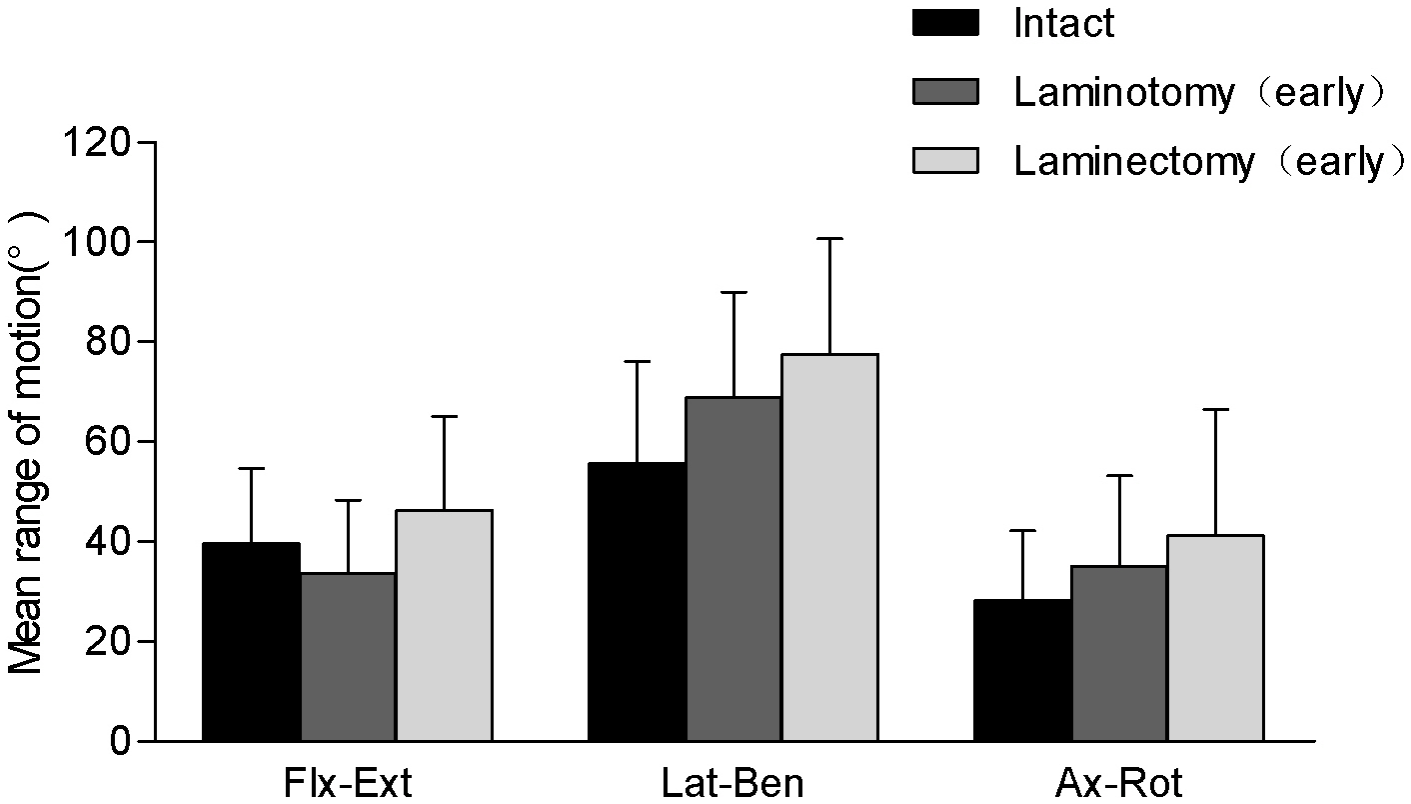
Initial differences between recapping laminoplasty and laminectomy. The range of motion (with one standard deviation error bar) for the INT, recapping laminoplasty (abbreviated as laminoplasty) and laminectomy conditions for the three rotation planes. There was no statistical difference (p>0.05) in motion for any condition.

At the immediate postoperative time, combined flexion–extension was not significantly different among the three conditions. Although the laminectomy group exhibited a nearly a 40% increase in combined lateral bending range of motion (77.6°±23.1), this difference was not statistically significant compared with the INT (55.7°±20.5) and the recapping laminoplasty (68.8°±21.1°) conditions. Finally, combined right and left axial rotation, intact (28.2°±14.0), recapping laminoplasty (35.0°±18.2), and laminectomy (41.2°±25.3) exhibit an increasing trend, there was no significant difference (Fig. 8).

## Discussion

The recapping laminoplasty procedure for surgical treatment of multilevel cervical entitative occupying lesions was developed in response to the steep demand of more extensive dural exposure and better postoperative stability. Recapping laminoplasty provides a sufficient surgical field and pursues a reconstruction of the whole posterior complex to minimize the impact on postoperative stability. This surgical procedure has been widely used, but currently available literatures talking about recapping laminoplasty are almost always short-term follow-up studies that have demonstrated better clinical outcomes and patient satisfaction [6], [7], [15], [16], [17]. To our best knowledge, no reports have demonstrated the anatomical changes and biomechanical effects of recapping laminoplasty.

The most notable morphological changes of the cervical spine after six months was the significantly volume reduction of the re-implanted lamina. The reason is obvious: the blood supply of the recapping lamina was completely cut off during surgery, and it can be only partially compensated in the process of healing. So, the insufficient blood supply can simply ensure the survival of a small amount of bone. However, there was no occurrence of osteoporosis in the surviving laminae, the bone density of the residual laminae were visually almost the same as the vertebral arch with no intervention. Secondly, bone nonunion was also found in this animal experiment [18]. Because of the nonunion of the bone section, there are three kinds of state of the re-capping laminas: bilateral fusion, unilateral fusion and fibrous connection only. Fibrous connection only can lead to the formation of pseudoarthrosis. The tips of the high-speed drill used in this experiment has a diameter of 1.5 mm. Application of smaller tips may increase the chance of healing, but the operation time will be obviously prolonged. Kawahara reported a 100% bony union rate in 24 patients using threadwire saw [8]. His recapping laminoplasty technique for spinal canal surgery is brilliant, but there is the risk of spinal cord injury, especially when the tumor and vascular malformation exist. In our operation, the dill gradually entered from the outside, so it was more secure. Thirdly, there were abundantly irregular fibrosis formation around the facet joints and re-implanted lamina-ligamentous complex. This may be a result of not only surgical stimulation but also body’s response to potential instability. Fibrous scar between vertebral joints can significantly limit the intervertebral motion. In short, factors that maintain the stability (bone nonunion and fibrosis formation) and the ones weaken stability (disappearance of the spinous processes, supraspinal and interspinous ligaments) exist simultaneously.

Nonetheless, generally speaking, recapping laminoplasty have better performances to maintain the stability of the cervical spine in the long term compared with laminectomy. And, these differences are mainly manifested in flexion-extension status. For the recapping laminoplasty and INT groups, although the average number was different, there was no statistical difference. But recapping laminoplasty and laminectomy was significantly different. We believe this was the result of the reconstruction of the posterior osteo-ligamentous complex. Ligament tissues can resist the power of flexion, and spinous processes can block each other to reduce the magnitude of extension. Moreover, we found there was appreciable abundant fibrous tissue formation along the two sides of re-implanted laminae. Specifically, fibrous tissue formation of the cervical spine after operation has been reported. Christian, using a caprine model, found postoperative fibrous tissue formation can significantly reduce the ROM of open door laminoplasty (ODL) and French door laminoplasty (FDL) techniques [19]. Clinically, one should expect that, in the long term, scar tissue formation in and around the facet joints and posterior lamina-ligamentous complex may limit intervertebral motion after cervical recapping laminoplasty. In addition, data that we are reporting for the laminectomy and INT groups agree with the afore-mentioned studies. Laminectomy can significantly increase the instability of cervical spine after surgery [20], [21], [22].

Unlike six months after surgery, there were no statistically significant differences in most loading conditions between recapping laminoplasty and laminectomy in the early postoperative state. Although the posterior osteo-ligamentous complex was destroyed or preserved in different surgery methods, more structures which are responsible to the stability of the spine remained undisturbed. Perhaps this is the reason why there were no significant changes in early postoperative stability. The important clinical implication is that stability and range of motion are not compromised in the immediate postoperative period. Besides, our research showed that laminectomy and INT groups had no differences immediately after operation, the data didn’t agree with some previous studies somewhat [23], [24]. It may because facet-sparing laminectomy induced a little kinematical change inherently in the immediate postoperative time point [25], [26]. Also, caprine vertebral canal’s diameter accounted for a small proportion of the vertebral body’s [27], [28]. So, caprine laminae had less impact on the stability of the entire spine and it was harder to come to a result of differences.

One of this project’s other aims was to investigate the biomechanical effect of adding progressively more levels to the recapping laminoplasty procedure. Our results showed that extending recapping laminoplasty from two to four segments did not cause significant differences in the immediate postoperative kinetics. The clinical significance is that when the surgeon plan to extend recapping laminoplasty procedures to adjacent levels, postoperative short-term stability should not be a major consideration. Moreover, postoperative external bracing can be waived, while early functional exercise and physical therapy can be implemented. This study is not without limitations, most of them attributable to the ex vivo nature of the investigation. Firstly, the motion of the specimens was simulated by pure moments imposed at both ends. It is different from the normal state of direct muscle force activation. And, we did not induce spinal cord tumors or vascular malformations in experimental animals which existed in clinical practice. However, as this is a comparative experiment, these two points may be not big issues. Furthermore, including too many factors inherently increase variability in the baseline condition. Secondly, inter-specimen variability is always a concern when performing animal studies. However, rigorous statistical analysis (and repeated-measure design, when allowable) allowed us to minimize these unavoidable effects. Thirdly, we only observed two time points (start and end), without describing the whole process of change. Nevertheless, Matthew et al have reported that progressive radiographic deformity does not occur at 6 months in a clinical trial [7].

## Conclusion

Overall, recapping laminoplasty can’t fully restore the posterior structure, but still reduced the risk of delayed cervical instability in a caprine model.
